# Prospective associations between early childhood mental health concerns and formal diagnosis of neurodevelopmental disorders in adolescence

**DOI:** 10.3389/fpsyt.2024.1356037

**Published:** 2024-09-18

**Authors:** Bright Opoku Ahinkorah, Christa Lam-Cassettari, James Rufus John, Valsamma Eapen

**Affiliations:** ^1^ School of Clinical Medicine, Discipline of Psychiatry & Mental Health, University of New South Wales, Sydney, NSW, Australia; ^2^ Academic Unit of Infant, Child, and Adolescent Psychiatry Services (AUCS), Ingham Institute of Applied Medical Research, Liverpool, NSW, Australia; ^3^ Academic Unit of Infant, Child, and Adolescent Psychiatry Services (AUCS), South Western Sydney Local Health District, Liverpool, NSW, Australia

**Keywords:** childhood, adolescence, longitudinal trajectory, mental health, neurodevelopmental disorders, social determinants of health

## Abstract

**Introduction:**

Understanding associations between psychosocial development in early childhood and formal diagnosis of neurodevelopmental disorders (NDDs) in adolescence is critical for early identification and for tailoring interventions and support. This study investigated whether the Strengths and Difficulties Questionnaire (SDQ) scores in early childhood (4-5 years) predict mental health (MH) problems as evidenced by SDQ scores and formal diagnosis of NDDs in adolescence (16-17 years).

**Methods:**

This study analysed data from a sample of 4968 children and adolescents using data from the Longitudinal Study of Australian Children. We used hierarchical regression models to determine the association between SDQ subscales and total scores at ages 4-5 years (primary exposure) and total SDQ scores and NDD diagnoses at ages 16-17 years (outcomes) whilst controlling for sociodemographic risk factors.

**Results:**

Each unit increase in SDQ score at age 4-5 led to a rise in SDQ scores at age 16-17. Autism and ADHD diagnoses, female gender, lower maternal education, and financial hardship were associated with higher SDQ scores at age 16-17. Furthermore, parent reported SDQ at age 4-5 was linked to higher likelihoods of formal diagnoses of ADHD, autism, and ADHD/autism at age 16-17. Additionally, social determinants of health such as female gender, culturally and linguistically diverse (CALD) backgrounds, and financial hardship were associated with increased odds of ADHD, autism, and ADHD/autism diagnoses at age 16-17.

**Conclusion:**

Our findings highlight the opportunity for early identification of transdiagnostic developmental and MH issues in the preschool period. Findings also emphasise the critical role of social determinants of health in the longitudinal trajectory of MH and NDDs and highlight the need for implementing early supports for improving peer relations and behavioural support strategies. If coupled with wrap around social care, early support strategies can enhance MH and wellbeing in adolescence and beyond.

## 1 Introduction

The increasing prevalence of mental health (MH) disorders among children is a significant challenge to both health and health care systems in Australia and worldwide (1, 2). International literature suggests that 80% of chronic MH disorders begin in childhood with 50% of MH problems established by 14 years of age (3). In Australia, one in five Australian children have a developmental vulnerability at the start of school (4), and it is estimated that about 14% of Australian children aged 4-11 years’ experience a MH disorder (5). However, 50% of children experiencing MH challenges are reported to be not receiving professional help (6) and often, identification of difficulties among these children does not occur until school commencement or later in adolescence, after complexities and comorbidities have occurred (7, 8). MH issues that emerge during these formative years can have profound and lasting effects on an individual’s life trajectory in terms of their future health, social functioning, and overall quality of life (9, 10). Besides health burden to children and families and its impact on adverse life trajectory, there are also significant economic ramifications with the cost of late intervention estimated to be $15.2 billion annually for high intensity and crisis services which are more expensive and less effective (11, 12). Therefore, early identification and intervention support in the first 2000 days of life could lead to significant improvement in children’s early life experiences, health, and overall development (13).

Standardised assessments are crucial to validate case definitions and enable the comparison of child MH concerns and risk factors over time. The Strengths and Difficulties Questionnaire (SDQ) (14) is a widely recognised tool for assessing behavioural and emotional problems in children and adolescents aged 4 to 16 years. It is a brief behavioural questionnaire that can be completed by teachers, parents, or adolescents, and comprises five subscales: emotional symptoms, conduct problems, hyperactivity/inattention, peer relationship problems, and prosocial behaviour. The SDQ has been used widely in community samples to not only measure common forms of psychopathology (15, 16) but has also proven valuable in predicting “real world” clinical outcomes such as formal diagnosis of neurodevelopmental disorders (NDDs) such as Autism Spectrum Disorder (ASD), Attention-Deficit/Hyperactivity Disorder (ADHD), among others (17, 18). This in turn proves invaluable in early identification of MH concerns of NDDs and informing interventions for supporting child development. We considered the SDQ for assessing mental health concerns because of its predictive validity, ease of use, multi-informant reports, strong normative data and comprehensive assessment of various emotional and behavioural domains (16, 19–22).

Previous research has established that childhood behaviours can serve as indicators of subsequent behavioural trends during adolescence and adulthood (23, 24). Despite the extensive body of literature exploring the predictive validity of childhood behavioural assessments, limited attention has been directed towards examining how individual subscales on routine childhood screeners in the preschool period relate to subsequent MH problems and formal diagnosis of NDDs in adolescence. Additionally, there is scarcity of evidence to determine the longitudinal prediction of SDQ from preschool age to adolescence. Further, studies have generally failed to account for key sociodemographic and sociocultural factors that shape the behavioural continuity and change from childhood to adolescence (25, 26) when assessing the predictive validity of childhood behavioural assessments.

To address the knowledge gap, this study used data from the Longitudinal Study of Australian Children (LSAC) to examine whether early childhood MH concerns longitudinally predict MH difficulties and formal NDD diagnosis in adolescence. Specifically, the objectives of this study were to (i) estimate the prevalence of MH problems in early childhood as measured by parent reported SDQ; (ii) determine the association between SDQ subscales and key social determinants of health during early childhood (age 4-5) and general MH issues as evidenced by total SDQ scores at adolescence (age 16-17); and (iii) determine the association between SDQ subscales and key social determinants of health during early childhood (age 4-5) and formal NDD diagnosis at adolescence (age 16-17). Findings from this study are expected to provide insights into the specific subscales that exert a stronger influence on the general MH as well as NDDs during adolescence, thereby guiding targeted interventions and support strategies to promote better life-trajectory outcomes.

## 2 Methods

### 2.1 Study design and data source

This study is a secondary analysis of the Longitudinal Study of Australian Children (LSAC) data, a nationally representative cohort of Australian households that gathers information about the development and welfare of children in areas such as parenting, family, peers, education, childcare, and health (27). The comprehensive data collected through repeated assessments across various developmental stages, offers a unique opportunity to discern nuanced trajectories of behavioural and emotional problems from childhood to adolescence. Data for nine waves have been collected to date from two representative cohorts of randomly sampled children. In LSAC, each wave of data is collected every two years. The cohorts are the birth (B) cohort and the kindergarten (K) cohort. The B cohort consists of infants born in March 2003 to February 2004 and the K cohort is made of children born in March 1999 to February 2000. The sampling frame for the B and K cohorts are 5,107 and 4,983 respectively. The study began with detailed interviews with parents on children’s development, social, economic and health issues. More than 98% of parents involved in the study were mothers. Starting from Wave 2 for the K cohort and Wave 4 for the B cohort, data were collected directly from the study children as well. For the purposes of this study, we focused on data from the B cohort that were collected in 2004 (Wave 1) and 2016 (Wave 7), where the children were aged 4-5 years and 16-17 years respectively. Wave 7 had a response rate of 62%, representing a sample of 3089.

### 2.2 Study measures

#### 2.2.1 The strengths and difficulties questionnaire (SDQ)

Comprises five subscales, which assess emotional problems, conduct problems, hyperactivity, peer problems, and pro-social behaviour. Each scale includes five items rated by the parent/carer and the study child (at age 16-17) as either: Never = 0, Somewhat True = 1 or Certainly True = 2. Responses to these items are scored on a scale from 0 to 10. These scales are aggregated, with the pro-social scale excluded, to calculate a ‘total difficulties’ score, ranging from 0 to 40 (28). For the parent report, SDQ total scores of 17 and above are considered abnormal. Clinical cut-off raw scores for the subscales (out of a possible 10) are: Emotion ≥ 5, Conduct ≥ 4, Hyperactivity ≥ 7, Peer Problems ≥ 4, Prosocial Behaviour ≤ 4 (29). For the study child report, SDQ total scores of 20 and above are considered abnormal. Clinical cut-off raw scores for the subscales (out of a possible 10) are: Emotion ≥ 7, Conduct ≥ 5, Hyperactivity ≥ 7, Peer Problems ≥ 6, Prosocial Behaviour ≤ 4 (21, 22). The LSAC data also adopts these five scales and assesses an overall SDQ score by aggregating scores from the emotional problems, conduct problems, hyperactivity, peer problems.

#### 2.2.2 Mental health and neurodevelopmental diagnosis

ASD and ADHD were selected as examples of NDD in this study with the MH problems assessed at age 16-17. In the LSAC, autism was assessed by asking Parent 1 (P1) the question “Does study child have any of these ongoing conditions? Autism, Asperger, or other autism spectrum”. For ADHD, P1 was asked “Does study child have any of these ongoing conditions? P1 is defined as the parent who knows the study child best; in most cases this is the child’s biological mother. ‘Ongoing conditions’ exist for some period of time (weeks, months or years) or re-occur regularly. They do not have to be diagnosed by a doctor). Each of these questions had a binary response (Yes/No).

#### 2.2.3 Covariates

Eight sociodemographic variables were included as controls or confounding variables: (1) Region of residence (metro and non-metro), (2) Indigenous status (indigenous and not indigenous), (3) sex (boy and girl), (4) mother’s level of education (Year 11 or less, Year 12, Post-Secondary, Bachelors, and Post-Graduate), and (5) culturally and linguistically diverse (CALD) status, which was created from the country of origin and language spoken at home of both Parent 1 and 2. Other controls were number of household members, number of siblings, and experience of financial hardship. Experience of financial hardship was measured by the study child’s household experiencing financial hardship in the previous twelve months (derived from seven items reported by the parent, for example not able to pay the mortgage or rent payments on time and sought assistance from a welfare or community organisation).

### 2.3 Analytical strategy

We analysed the data using Stata version 17 (30). Longitudinal weights were applied to enhance representativeness concerning the Australian population aged 4-5 and 16-17 years (31). For sample characteristics, we computed descriptive statistics, including frequencies, percentages, means, and proportions, for all variables at both 4-5 and 16-17 age groups. We also examined the gender differences in abnormal SDQ scores using chi-square test of independence. Multivariable hierarchical linear regression analysis was conducted to examine the association between P1 report of SDQ scores at age 4-5 and P1 and child reports of SDQ scores at age 16-17. This analysis included two outcomes. Firstly, we examined P1 report of SDQ subscales at 4-5 years as predictors and P1 report of total SDQ at 16-17 years. Similarly, we developed models with P1 report of SDQ subscales at 4-5 years as predictors and study child’s report of total SDQ at 16-17 years. Three models were computed for both outcomes: Model 1 - SDQ subscales at age 4-5 only; Model 2 – SDQ subscales at age 4-5 + MH diagnosis variables; Model 3 – SDQ subscales at age 4-5 + MH diagnosis variables + sociodemographic covariates. Finally, we also used multivariable hierarchical binary logistic regression to examine the association between SDQ at age 4-5 and MH diagnoses at age 16-17 (ADHD, Autism, multiple MH disorders). For all three outcomes, two models were employed: Model 1 - SDQ subscales at 4-5 years only; Model 2 – SDQ subscales at age 4-5 + sociodemographic covariates.

## 3 Results

### 3.1 Sample characteristics

**Table 1** shows the sociodemographic and MH diagnosis characteristics of the respondents. Few of the study children had autism (3.5%) and ADHD (3.0%). Most of the respondents lived in metropolitan areas, were non-indigenous, had male children, and had post-secondary education or higher. Most of the respondents lived in households of 4-6 members, had children with one or more siblings, were non-CALD, and never experienced financial hardship.

**Table 1:** Mental health condition and sociodemographic characteristics.

Variables	Age 4/5		Age 16/17	
	N	%	N	%
Multiple MH (at age 16)				
*No*	NA	NA	2852	94.2
*Yes*	NA	NA	176	5.8
*Total*	NA	NA	3028	100.0
Autism/Asperger/autism spectrum (at age 16)				
*No*	NA	NA	2921	96.5
*Yes*	NA	NA	107	3.5
*Total*	NA	NA	3028	100.0
Attention Deficit Disorder (at age 16)				
*No*	NA	NA	2937	97.0
*Yes*	NA	NA	91	3.0
*Total*	NA	NA	3028	100.0
Residential Location				
*Metropolitan*	3174	63.7	1899	61.5
*Non-metropolitan*	1808	36.3	1190	38.5
*Total*	4983	100.0	3089	100.0
Indigenous status				
*Not indigenous*	4787	96.1	3002	97.2
*Indigenous*	194	3.9	87	2.8
*Total*	4981	100.0	3089	100.0
Sex				
*Male*	2553	51.2	1588	51.4
*Female*	2430	48.8	1501	48.6
*Total*	4983	100.0	3089	100.0
P1 Highest Education				
*Year 11 or less*	1316	26.4	474	15.6
*Year 12*	613	12.3	198	6.5
*Post-Secondary*	1849	37.1	1447	47.7
*Bachelors*	670	13.5	428	14.1
*Post-Graduate*	530	10.7	491	16.2
*Total*	4978	100.0	3038	100.0
No. people in household				
*2-3*	767	15.4	706	23.3
*4-5*	3437	69.0	1859	61.3
*6 or more*	779	15.6	469	15.5
*Total*	4983	100.0	3034	100.0
No. siblings of study child in household				
*0*	572	11.5	503	16.6
*1*	2369	47.5	1320	43.5
*2 or more*	2042	41.0	1211	39.9
*Total*	4983	100.0	3034	100.0
Family CALD Status				
*Non-CALD*	3817	76.8	2417	80.4
*CALD*	1150	23.2	591	19.6
*Total*	4968	100.0	3008	100.0
Hardship scale				
*No hardship*	3362	67.7	2682	90.2
*Experienced hardship*	1607	32.3	291	9.8
*Total*	4969	100.0	2973	100.0

Overall, the mean SDQ total score at both ages 4-5 and 16-17 indicate a normal SDQ among the study children (see **Table 2**). At age 4-5, 11.5% of the 4968 study children had an abnormal total SDQ score. Conduct problems contributed more to the abnormal total SDQ score (28.8%) in preschoolers. At age 16-17, total abnormal SDQ score was reported by 10.1% of the study children and 9.0% of mothers. Emotional problems contributed more to the total abnormal SDQ score at age 16-17. This varied from 15.2% from the child’s report to 17% from Parent 1 reports (**Table 2**). When the results were segregated by gender and their associations tested using a chi-square test of independence, the results showed that males have significantly higher abnormal SDQ scores compared to females at age 4-5. However, at age 16-17, males had a significantly higher scores compared to females only for externalising behaviours (hyperactivity and conduct problems), whiles females had a significantly higher SDQ scores for internalising behaviours (emotional problems) compared to males (see **Supplementary Table S1**).

**Table 2:** Descriptive statistics of SDQ Scales at age 4/5 and 16/17 (parent and child reports).

Variable	N	Abnormal n (%)	Mean (SD)
Parent-report			
P1 report of SDQ Prosociality at age 4/5	4,969	87 (1.7)	7.7 (1.8)
P1 report SDQ Hyperactivity scale at age 4/5	4,969	550 (11.1)	3.6 (2.3)
P1 report of SDQ Emotional symptoms at age 4/5	4,968	399 (8.0)	1.7 (1.7)
P1 report of SDQ Peer problems scale at age 4/5	4,969	666 (13.4)	1.7 (1.6)
P1 report of SDQ Conduct problems scale at age 4/5	4,969	1433 (28.8)	2.5 (2.0)
Total SDQ score based on P1 report of subscales at age 4/5	4,968	572 (11.5)	9.6 (5.4)
P1 report SDQ Prosociality scale at age 16/17	2,999	48 (1.6)	8.1 (1.8)
P1report of SDQ Hyperactivity scale at age 16/17	2,998	152 (5.1)	2.6 (2.2)
P1report of SDQ Emotional problems scale at age 16/17	2,998	508 (17.0)	2.3 (2.2)
P1report of SDQ Peer problems scale at age 16/17	2,998	460 (15.4)	1.6 (1.7)
P1report of SDQ Conduct problems at age 16/17	2,998	209 (7.0)	1.1 (1.4)
Total SDQ score based on P1 report of subscales at age 16/17	2,998	271 (9.0)	7.4 (5.6)
Study child report			
SC report SDQ Prosociality scale at age 16/17	2,947	47 (1.6)	7.7 (1.7)
SC report of SDQ Hyperactivity scale at age 16/17	2,947	484 (16.4)	4.1 (2.3)
SC report of SDQ Emotional problems scale at age 16/17	2,946	448 (15.2)	3.4 (2.5)
SC report of SDQ Peer problems scale at age 16/17	2,947	127 (4.3)	2.0 (1.7)
SC report of SDQ Conduct problems at age 16/17	2,947	211 (7.2)	1.7 (1.6)
Total SDQ score based on SC report of subscales at age 16/17	2,946	297 (10.1)	11.2 (5.8)

P1, Parent 1; SC, Study Child.

### 3.2 Relationship between parent report of SDQ at age 4-5 and SDQ at 16-17

**Table 3** displays results on the relationship between parent report of SDQ at age 4-5 and SDQ at 16-17. The results indicate that for every unit increase in parent report of total SDQ score at age 4-5, we expect a 0.47 (Model 1), 0.44 (Model 2) and 0.38 (Model 3) point increase in total SDQ score at age 16-17. Consistent results were obtained for the SDQ hyperactivity, emotional symptoms, peer problems, and conduct problems subscales. Having Autism and ADHD contributed to an increase in SDQ scores at 16-17. Female children had higher SDQ scores compared to male children. Higher levels of mother’s level of education decreased SDQ scores at 16-17. Finally, experience of financial hardship contributed to an increase in SDQ scores at 16-17.

**Table 3:** Relationship between parent report of SDQ at age 4 and SDQ at 16/17.

Explanatory variables	Model 1 Coefficients (95% CI)	Model 2 Coefficients (95% CI)	Model 3 Coefficients (95% CI)
SDQ Prosociality scale	0.03 (-0.09,0.16)	-0.07 (-0.15,0.19)	0.03 (-0.09,0.15)
SDQ Hyperactivity scale	0.52 (0.42,0.62)^***^	0.36 (0.27,0.45)^***^	0.33 (0.24,0.43)^***^
Emotional symptoms scale	0.27 (0.12,0.41)^***^	0.28 (0.14,0.42)^**^	0.26 (0.12,0.40)^***^
Peer problems scale	0.60 (0.45,0.75)^***^	0.49 (0.35,0.63)^***^	0.45 (0.31,0.58)^***^
Conduct problems scale	0.50 (0.36,0.63)^***^	0.49 (0.37,0.61)^***^	0.45 (0.35,0.59)^***^
Total SDQ scale	0.47 (0.43,0.52)^***^	0.40 (0.36,0.44)^***^	0.37 (0.33,0.41)^***^
Autism/Asperger/autism spectrum (at age 16)			
*No*		Reference	Reference
*Yes*		6.02 (4.71,7.32)^***^	6.02 (4.73,7.32)^***^
Attention Deficit Disorder (at age 16)			
*No*		Reference	Reference
*Yes*		6.55 (5.30,7.80)^***^	6.62 (5.40,7.84)^***^
Study child’s age in months			-0.02 (-0.08,0.05)
Metro status			
*Metro*			Reference
*Non-metro*			-0.27 (-0.66,0.11)
Indigenous status			
*Not indigenous*			Reference
*Indigenous*			-0.42 (-1.67,0.83)
Sex			
*Male*			Reference
*Female*			0.68 (0.31,1.05)^***^
P1 Highest Education			
*Lower 2nd or less*			Reference
*Upper 2nd*			-0.41 (-1.11,0.29)
*Post-Secondary*			-0.64 (-1.20,-0.08)^*^
*Bachelors*			-1.31 (-1.93,-0.68)^***^
*Post-Graduate*			-1.12 (-1.80,-0.44)^**^
No. people in household			
*2-3*			Reference
*4-6*			-0.69 (-1.62,0.24)
*7 or more*			-1.03 (-2.13,0.06)
No. siblings of study child in household			
*0*			Reference
*1*			-0.68 (-1.72,0.35)
*2 or more*			-0.53 (-1.64,0.57)
CALD status			
*Non-CALD*			Reference
*CALD*			-0.23 (-0.71,0.25)
Hardship scale			
*No hardship*			Reference
*Experienced hardship*			1.01 (0.54,1.47)^***^
*N*	2993	2980	2967

95% confidence intervals in brackets.

^*^p < 0.05, ^**^p < 0.01, ^***^p < 0.001.

### 3.3 Relationship between parent’s report of SDQ at age 4-5 and child’s report at 16-17

**Table 4** shows results on the relationship between parent report of SDQ at age 4 and child’s report at 16-17. The results indicate that for every unit increase in parent report of total SDQ score at age 4-5, we expect a 0.20 (Model 1), 0.18 (Model 2) and 0.16 (Model 3) point increase in child report of total SDQ score at age 16-17. Consistent results were obtained for the SDQ hyperactivity, peer problems, and conduct problems subscales. Children who were diagnosed of Autism and ADHD had higher SDQ scores at 16-17. Both autism and ADHD had positive association with SDQ scores at 16-17. Female children had higher SDQ scores compared to male children. Higher levels of maternal education were associated with a decrease in SDQ scores at 16-17. Children in families with CALD background had lower SDQ scores. Finally, experience of financial hardship contributed to an increase in SDQ scores at 16-17.

**Table 4:** Relationship between parent’s report of SDQ at age 4 and child’s report at 16/17.

Explanatory variables	Model 1 Coefficients (95% CI)	Model 2 Coefficients (95% CI)	Model 3 Coefficients (95% CI)
SDQ Prosociality scale	0.08 (-0.06,0.22)	0.12 (-0.02,0.26)	0.06 (-0.08,0.20)
SDQ Hyperactivity scale	0.16 (0.05,0.28)^**^	0.11 (-0.01,0.22)	0.10 (-0.02,0.21)
Emotional symptoms scale	0.02 (-0.13,0.18)	0.03 (-0.13,0.18)	-0.01 (-0.15,0.15)
Peer problems scale	0.28 (0.11,0.44)^***^	0.24 (0.08,0.40)^**^	0.22 (0.06,0.38)^**^
Conduct problems scale	0.36 (0.23,0.49)^***^	0.37 (0.24,0.50)^***^	0.33 (0.20,0.46)^***^
Total SDQ scale	0.20 (0.16,0.25)^***^	0.18 (0.13,0.22)^***^	0.16 (0.11,0.21)^***^
Autism/Asperger/autism spectrum (at age 16)			
*No*		Reference	Reference
*Yes*		2.21 (0.95,3.47)^***^	2.33 (1.09,3.56)^***^
Attention Deficit Disorder (at age 16)			
*No*		Reference	Reference
*Yes*		2.92 (1.55,4.29)^***^	3.11 (1.77,4.46)^***^
Study child’s age in months			-0.08 (-0.16,-0.00)
Metro status			
*Metro*			Reference
*Non-metro*			-0.14 (-0.60,0.31)
Indigenous status			
*Not indigenous*			Reference
*Indigenous*			-0.48 (-1.98,1.02)
Sex			
*Male*			Reference
*Female*			1.61 (1.17,2.05)^***^
P1 Highest Education			
*Lower 2nd or less*			Reference
*Upper 2nd*			0.37 (-0.41,1.14)
*Post-Secondary*			-0.14 (-0.79,0.50)
*Bachelors*			-1.37 (-2.09,-0.65)^***^
*Post-Graduate*			-0.91 (-1.68,-0.13)^*^
No. people in household			
*2-3*			Reference
*4-6*			-0.83 (-2.06,0.41)
*7 or more*			-0.69 (-2.05,0.67)
No. siblings of study child in household			
*0*			Reference
*1*			-0.14 (-1.29,1.06)
*2 or more*			0.52 (-0.75,1.78)
CALD status			
*Non-CALD*			Reference
*CALD*			-0.81 (-1.35,-0.26)^**^
Hardship scale			
*No hardship*			Reference
*Experienced hardship*			1.20 (0.66,1.73)^***^
*N*	2941	2889	2876

95% confidence intervals in brackets.

^*^p < 0.05, ^**^p < 0.01, ^***^p < 0.001.

### 3.4 Association between parent’s report of SDQ at age 4-5 and MH diagnoses

**Table 5** shows results on the relationship between parent report of SDQ at age 4 and MH diagnoses. The results show that higher total SDQ score at age 4-5 increased the likelihood of MH diagnoses at age 16-17. SDQ Hyperactivity contributed to an increase in ADHD, autism, and ADHD/autism. SDQ prosociality increased the likelihood of autism only and peer problems increased the likelihood of autism and ADHD/autism. Female children had higher likelihood of MH diagnoses compared to male children. Children from CALD background were more likely to experience MH diagnoses compared to those from non-CALD background. The experience of financial hardship contributed to an increase in autism and ADHD/autism at age 16-17.

**Table 5:** Relationship between SDQ subscales and neurodevelopmental disorders.

Explanatory variables	ADHD		Autism		Neurodevelopmental disorder	
	Model 1 AOR (95% CI)	Model 2 AOR (95% CI)	Model 1 AOR (95% CI)	Model 2 AOR (95% CI)	Model 1 AOR (95% CI)	Model 2 AOR (95% CI)
SDQ Prosociality scale	1.06	1.08	0.86^*^	0.86^*^	0.94	0.95
	(0.91,1.23)	(0.93,1.26)	(0.75,0.98)	(0.75,0.99)	(0.84,1.05)	(0.85,1.06)
SDQ Hyperactivity scale	1.52^***^	1.48^***^	1.40^***^	1.36^***^	1.46^***^	1.42^***^
	(1.36,1.70)	(1.33,1.65)	(1.25,1.58)	(1.20,1.53)	(1.33,1.59)	(1.30,1.55)
Emotional symptoms scale	0.96	0.95	1.01	1.01	1.00	0.99
	(0.84,1.10)	(0.83,1.09)	(0.88,1.17)	(0.86,1.17)	(0.90,1.11)	(0.89,1.10)
Peer problems scale	0.99	1.01	1.43^***^	1.46^***^	1.25^***^	1.29^***^
	(0.87,1.13)	(0.89,1.16)	(1.26,1.62)	(1.28,1.67)	(1.13,1.39)	(1.16,1.43)
Conduct problems scale	1.08	1.07	0.98	0.96	1.03	1.01
	(0.95,1.23)	(0.94,1.22)	(0.85,1.13)	(0.82,1.12)	(0.93,1.13)	(0.90,1.12)
Total SDQ scale	1.15^***^	1.14^***^	1.22^***^	1.20^***^	1.19^***^	1.18^***^
	(1.10,1.19)	(1.09,1.18)	(1.18,1.26)	(1.16,1.24)	(1.16,1.23)	(1.14,1.22)
Study child’s age in months		1.09^*^		0.99		1.02
		(1.01,1.19)		(0.89,1.09)		(0.95,1.10)
Metro status						
*Metro*		Reference		Reference		Reference
*Non-metro*		0.72		0.67		0.69
		(0.44,1.19)		(0.41,1.10)		(0.48,1.01)
Indigenous status						
*Not indigenous*		Reference		Reference		Reference
*Indigenous*		1		0.75		0.38
		(1.00,1.00)		(0.12,4.63)		(0.08,1.96)
Sex						
*Male*		Reference		Reference		Reference
*Female*		0.41^**^		0.36^***^		0.43^***^
		(0.23,0.71)		(0.21,0.63)		(0.28,0.64)
P1 Highest Education						
*Lower 2nd or less*		Reference		Reference		Reference
*Upper 2nd*		1.21		1.07		1.30
		(0.55,2.67)		(0.48,2.37)		(0.70,2.39)
*Post-Secondary*		1.22		1.08		1.38
		(0.63,2.35)		(0.61,2.27)		(0.84,2.29)
*Bachelors*		1.44		1.31		1.58
		(0.67,3.09)		(0.60,2.90)		(0.86,2.88)
*Post-Graduate*		1.59		1.01		1.58
		(0.65,3.85)		(0.39,2.57)		(0.78,3.17)
No. people in household						
*2-3*		Reference		Reference		Reference
*4-6*		1.35		1.00		1.13
		(0.59,3.11)		(0.43,2.32)		(0.59,2.14)
*7 or more*		2.31		1.27		1.88
		(0.81,6.58)		(0.42,3.86)		(0.84,4.22)
No. siblings of study child in household						
*0*		Reference		Reference		Reference
*1*		0.60		1.13		0.86
		(0.25,1.42)		(0.46,2.78)		(0.43,1.68)
*2 or more*		0.51		0.59		0.55
		(0.19,1.39)		(0.21,1.63)		(0.26,1.18)
CALD status						
*Non-CALD*		Reference		Reference		Reference
*CALD*		0.28^**^		0.35^**^		0.33^***^
		(0.11,0.70)		(0.17,0.74)		(0.19,0.60)
Hardship scale						
*No hardship*		Reference		Reference		Reference
*Experienced hardship*		1.52		1.69^*^		1.73^**^
		((0.91,2.51)		(1.05,2.72)		(1.19,2.52)
*N*	3021	3008	3021	3008	3021	3008

AOR, Adjusted odds ratio; Exponentiated coefficients; 95% confidence intervals in brackets.

^*^p < 0.05, ^**^p < 0.01, ^***^p < 0.001.

## 4 Discussion

This study examined the prevalence of MH concerns in early childhood using the widely used SDQ survey in a sample of 4968 children and adolescents from the LSAC data Birth cohort. The results indicated that the mean SDQ total score for almost 90% of the study children were within the ‘normal’ range in the preschool years (ages 4-5) and again in late adolescence (ages 16-17). At age 4-5, 11.5% of the cohort had an abnormal total SDQ score. Conduct problems appeared to contribute most to the abnormal total SDQ score, with peer problems the next largest contributor. By the time children reached late adolescence (ages 16-17 years), an abnormal total SDQ score was reported by 9.0% of the study children and 20.1% of the reporting parent. Emotional problems appeared to contribute most to the total abnormal SDQ score, with peer problems the second largest contributor to an abnormal total SDQ score as reported by the adolescent and the reporting parent. These findings concur with previous evidence that children who exhibit MH concerns during the preschool years are more likely to have an increased risk of mental disorders in adolescence (32, 33).

During early childhood, children often experience heightened peer problems and conduct issues due to their ongoing development in social skills, emotional regulation, and exposure to new social environments such as preschool (34, 35). At this stage, they begin learning to explore social interactions, which can lead to conflicts, peer rejection, and behavioural challenges like impulsivity or aggression. Environmental factors such as family dynamics and peer influences also play significant roles in shaping these behaviours. As children mature into adolescence (ages 16/17), they typically exhibit a reduction in these issues. This transition is influenced by improved emotional regulation, social skills acquisition, and increased maturity through social experiences (35). There are however gender differences in both internalising and externalising behaviours. As found in our study, males have significantly higher abnormal SDQ scores compared to females at age 4-5. However, at adolescence, boys tend to have higher abnormal values in externalising problems, while girls exhibit higher internalising problems. The possible reasons for this could be both psychological and social. Psychologically, during adolescence, boys and girls are typically in the process of forming their identities, which can be a source of stress and contribute to both internalising and externalising behaviours (36, 37). Boys may externalise this stress through aggressive or disruptive behaviour, while girls may internalise it, leading to emotional issues. Socially, peer relationships become increasingly important during adolescence. Boys often engage in competitive and sometimes aggressive interactions, which can lead to externalising behaviours. Girls, on the other hand, may experience relational aggression and social stress, contributing to internalising behaviours (36, 37).

The second objective of the study was to identify the association between SDQ measures of MH problems in early childhood and later adolescence. Consistent with other studies (32, 33), the current results showed continuity in heighted SDQ scores. That is, those with a higher SDQ total score at age 4-5 were more likely to have higher SDQ total score at age 16-17 years. Our findings that lower levels of maternal education, and increased financial hardship are associated with higher SDQ scores in childhood is in keeping with previous studies (38), and provides new evidence that this association continues into adolescence. Unlike previous studies (39), CALD status was not associated with increased SDQ total scores at years, although there was a positive association during the preschool age.

This study examined the association between MH problems as measured by the SDQ in early childhood and formal diagnosis of NDD at adolescence. In keeping with the findings from previous studies (17, 18, 23, 24), our results showed that children with higher total SDQ scores in the preschool years were more likely to have a diagnosed MH condition in adolescence. Further, this shows evidence that self-reporting by adolescents and parental reports on the SDQ are consistent. Given that the SDQ is an easy-to-use self-report tool, this provides further evidence that there is utility in providing the SDQ for screening in community samples. This also adds to the knowledge base that SDQ is a valuable tool for the identification of core behavioural symptoms via the subscales and can assist in profiling the characteristics of behavioural and emotional problems in children with confirmed MH and NDDs (40).

Closer inspection of the sociodemographic risk factors associated with adolescents’ MH concerns showed that female gender (compared to male), primary caregiver’s level of education, financial hardship, and CALD status were not only associated with SDQ scores but also linked to formal diagnoses of NDDs at age 16-17. The protective effect among female children is consistent with Lawrence et al’s Australian child and adolescent survey of mental health and wellbeing report where (5) males are more likely to have any MH disorder across childhood and adolescence compared to females, although during adolescence, females are more likely to experience anxiety or depression than males. It is important to note that the effect of gender on MH disorders may not be direct, but through proxy measures such as academic stress, bullying, peer relationship issues, parental mental health, parenting styles, and socio-economic status. Similarly, higher level of parental education was associated with lower SDQ scores as well as less likelihood of having any MH disorder or NDDs which is also consistent with several studies (41, 42). This may be due to reasons that higher level of parental education influences childrearing practices whilst also making better use of existing resources and obtain more up-to-date information regarding child health and wellbeing. On the other hand, we also found that financial hardship was associated with higher SDQ scores as well as higher risk of having a MH disorder or NDD. Low income levels are associated with education and employment as previously explained and reported in other LSAC studies (41, 43). Despite CALD status showing a negative association with child reports of MH concerns, it may be the case that cultural beliefs or stigma around MH seeking behaviours result in adolescents under reporting MH concerns on self-report measures (44, 45). This reiterates the need to take into account key sociodemographic and sociocultural risk factors when tailoring community awareness campaigns and population health programs to improve MH outcomes and reduce health system burden.

### 4.1 Implications for early intervention and policy

Including screening of mental health in early childhood can be useful in identifying children at risk of developing mental health issues so that targeted supports can be provided to improve long-term outcomes. Children diagnosed with ADHD, autism, or those exhibiting higher SDQ scores identifies children who may benefit from early and targeted support. Recognising that children from diverse backgrounds (e.g., CALD) and those experiencing financial hardship are more likely to have higher SDQ scores, interventions should be culturally sensitive and tailored to address specific socioeconomic challenges. This would ensure that interventions are effective and accessible. There is the need for educational programs to train parents, teachers, and caregivers to recognise early signs of mental health issues and understand the relevance of early intervention. Policies must address disparities in access to mental health services, especially for families with lower maternal education and those facing financial hardships. Providing equitable access would ensure that all children, regardless of their background, obtain the support they need.

### 4.2 Strengths and limitations

Strengths of this study include the large number of children and adolescents sampled as part of a population level study in Australia, as well as a high completion rate. In terms of the analysis, the study draws its strength from the use of a complex multivariable modelling. We also conducted a sensitivity analysis using the MH variables as outcome indicators, which shows the robustness of the analysis. The key limitations are the nature of the survey data in that it is observational in nature. Hence it is not possible to draw conclusions about the cause-and-effect relationship between formal diagnoses of NDDs and MH issues in early infancy. While associations between higher SDQ scores and later mental health outcomes can be observed, it is not possible to definitively conclude that the former causes the latter. Further, the SDQ scores and MH factors may have been over- or underreported because they were derived from parental and child self-reports. This has the potential in bringing bias to the data and the results obtained from the study. The SDQ is a concise tool used to assess MH. Although it has strong psychometric qualities, this study raises questions regarding its applicability to children who are significantly different in age. Bøe and Hysing (46), for instance, queried the appropriateness of an item measuring the frequency of tantrums among older adolescents. Since this behaviour is more typical in 4- and 5-year-olds than in 16 and 17 year olds, comparisons of total SDQ scores through time (as well as the age-related decline in total SDQ) must be interpreted with caution. Lastly, we must consider the representativeness of the sample, the effect of survey attrition, and item non-response, just like we do with any survey data. When compared to the 2001 Census, the original sample was fairly representative of the entire Australian population; nevertheless, it was marginally under representative of single-parent, non-English speaking, and rental property-dwelling families. These same traits have been linked to attrition over an extended period of time (31). At Wave 1 of LSAC, there were 4,983 children, but the final model’s analytic sample consisted of 2876. The differences in sample could limit the representativeness of the results.

## 5 Conclusion

This study provides further evidence that the SDQ subscales have the potential to identify the presence of MH concerns during early childhood and late adolescence. Specifically, it highlights the role of conduct and peer problems as the largest contributor to higher SDQ scores in adolescence. Hyperactivity and peer problems were the major contributor to the likelihood of diagnosis of NDDs in adolescence. Further, psychosocial factors such as cultural diversity and financial hardship were important social determinants to MH problems and NDDs in early childhood and adolescence respectively. These findings flag the importance of promoting positive peer relationships as well as social care support strategies starting from preschool period to improve behavioural trajectories in adolescence, which in turn can have a positive impact on the life course.

## Data Availability

The original contributions presented in the study are included in the article/**Supplementary Material**, further inquiries can be directed to the corresponding author/s.
