# Supplementary material for: Prospective associations between early childhood mental health concerns and formal diagnosis of neurodevelopmental disorders in adolescence
Source: Front Psychiatry. 2024 Sep 18;15:1356037. doi: 10.3389/fpsyt.2024.1356037 (PMC11445126; doi:10.3389/fpsyt.2024.1356037)
Supplement: Supplementary file 1 [file Table1.docx]

**Table S1: Gender differences in abnormal SDQ scores at age 4/5 and 16/17**

|  | **Abnormal SDQ** | | | |
| --- | --- | --- | --- | --- |
|  | **Male** | **Female** | **χ2** | **p-value** |
| **Parent-report** |  |  |  |  |
| P1 report of SDQ Prosociality at age 4/5 | 2.34 | 1.02 | 12.87 | <0.001 |
| P1 report SDQ Hyperactivity scale at age 4/5 | 14.17 | 6.88 | 69.84 | <0.001 |
| P1 report of SDQ Emotional symptoms at age 4/5 | 7.37 | 7.41 | 0.01 | 0.954 |
| P1 report of SDQ Peer problems scale at age 4/5 | 15.08 | 9.86 | 30.88 | <0.001 |
| P1 report of SDQ Conduct problems scale at age 4/5 | 30.56 | 25.21 | 17.64 | <0.001 |
| Total SDQ score based on P1 report of subscales at age 4/5 | 13.19 | 7.86 | 37.31 | <0.001 |
| P1 report SDQ Prosociality scale at age 16/17 | 2.10 | 0.95 | 6.50 | 0.011 |
| P1report of SDQ Hyperactivity scale at age 16/17 | 7.14 | 2.04 | 44.16 | <0.001 |
| P1report of SDQ Emotional problems scale at age 16/17 | 11.14 | 19.90 | 44.08 | <0.001 |
| P1report of SDQ Peer problems scale at age 16/17 | 14.15 | 13.38 | 0.38 | 0.54 |
| P1report of SDQ Conduct problems at age 16/17 | 6.88 | 5.37 | 2.98 | 0.084 |
| Total SDQ score based on P1 report of subscales at age 16/17 | 8.32 | 7.34 | 1.01 | 0.316 |
| **Study child report** |  |  |  |  |
| SC report SDQ Prosociality scale at age 16/17 | 2.07 | 0.83 | 7.92 | 0.005 |
| SC report of SDQ Hyperactivity scale at age 16/17 | 17.43 | 15.17 | 2.76 | 0.097 |
| SC report of SDQ Emotional problems scale at age 16/17 | 7.22 | 21.86 | 127.89 | <0.001 |
| SC report of SDQ Peer problems scale at age 16/17 | 3.87 | 3.79 | 0.01 | 0.909 |
| SC report of SDQ Conduct problems at age 16/17 | 8.55 | 4.34 | 21.49 | <0.001 |
| Total SDQ score based on SC report of subscales at age 16/17 | 7.82 | 10.48 | 6.29 | 0.012 |
